# Functional maturation of human neural stem cells in a 3D bioengineered brain model enriched with fetal brain-derived matrix

**DOI:** 10.1038/s41598-019-54248-1

**Published:** 2019-11-29

**Authors:** Disha Sood, Dana M. Cairns, Jayanth M. Dabbi, Charu Ramakrishnan, Karl Deisseroth, Lauren D. Black, Sabato Santaniello, David L. Kaplan

**Affiliations:** 1 Department of Biomedical Engineering, Tufts University, Medford, MA 02155 USA; 2 Department of Bioengineering, Stanford University, Stanford, CA 94305 USA; 3 Neural Systems Engineering and Control Laboratory, University of Connecticut, CT, 06269 USA

**Keywords:** Neural stem cells, Neurological models, Bioinspired materials, Tissue engineering

## Abstract

Brain extracellular matrix (ECM) is often overlooked *in vitro* brain tissue models, despite its instructive roles during development. Using developmental stage-sourced brain ECM in reproducible 3D bioengineered culture systems, we demonstrate enhanced functional differentiation of human induced neural stem cells (hiNSCs) into healthy neurons and astrocytes. Particularly, fetal brain tissue-derived ECM supported long-term maintenance of differentiated neurons, demonstrated by morphology, gene expression and secretome profiling. Astrocytes were evident within the second month of differentiation, and reactive astrogliosis was inhibited in brain ECM-enriched cultures when compared to unsupplemented cultures. Functional maturation of the differentiated hiNSCs within fetal ECM-enriched cultures was confirmed by calcium signaling and spectral/cluster analysis. Additionally, the study identified native biochemical cues in decellularized ECM with notable comparisons between fetal and adult brain-derived ECMs. The development of novel brain-specific biomaterials for generating mature *in vitro* brain models provides an important path forward for interrogation of neuron-glia interactions.

## Introduction

Advances in 3D *in vitro* organoid and spheroid-based systems using human induced pluripotent stem cells (hiPSCs) or human neural stem cells (hNSCs) have been extremely useful for studies of normal brain development, such as cortical layering/interneuron migration, and for deciphering pathological features underlying neurodevelopmental disorders such as microcephaly, lissencephaly, and autism^1–10^. Despite the variety of approaches there are only a few examples of 3D *in vitro* brain-like tissue models that exhibit co-differentiation into neurons and glial cell types^9–11^. Also, most of these models indicate slow differentiation into neuronal supporting cell types, such as astrocytes, and/or necrosis at longer time points of cultivation *in vitro*^12,13^. This co-differentiation into astrocytes and cell-cell interactions are key for revealing the molecular basis for many diseased states, particularly neurodegenerative disorders where neuron-glia interactions are dysfunctional^14–17^. Many of these 3D brain-like tissue models are also limited in terms of reproducibility, and compartmentalization related to the introduction of microglia and vasculature, as well as for sampling and control of nutrient transport into and out of the tissue systems.

A common limitation of current 3D *in vitro* brain models is that the ECM content is often not considered in detail, even though brain ECM is dynamic during development and plays a crucial role in cell signaling and homeostasis^18^. The ‘dynamic reciprocity’ model was proposed in the 1980s, which suggested that ECM guides gene expression and individual components of ECM have an instructive role in directing tissue-specific development^19^. Despite these roles, most 3D brain tissue models use Matrigel as the major ECM component and/or soluble bioactive factors to induce differentiation. Matrigel is a mouse sarcoma-derived basement membrane matrix that lacks many physiologically-relevant biochemical cues involved in brain development and maintenance, including several glycoproteins and proteogylcans^20–22^. The human brain ECM constitutes about 20–40% of the brain volume during development and adulthood, is highly organized, and has unique traits in composition when compared to the ECM of other tissues^18^. Moreover, during development, ECM guides the compartmentalization of functional brain microdomains, and thus contributes to the sophisticated architecture and function of the brain^23^. Such native ECM signals are particularly important for differentiation of neural progenitor/stem cells^24^.

The impact of some specific brain-ECM constituents such as laminin^25,26^ and adult brain-derived ECM on cell differentiation, synapse formation and mechanical properties has been studied in isolation^27–29^; however, the study of composite, scaffold-based 3D *in vitro* systems to investigate the bioactivity of ECM from different developmental stages over long-term differentiation of human induced neural stem cells (hiNSCs) into both neurons and astrocytes is lacking. Astrocytes respond to soluble factors and also influence their environment through the secretion of ECM molecules, particularly chondroitin sulfate proteoglycans (CSPGs) that vary with mature/resting versus reactive astrocytes^15,30,31^. Therefore, preventing reactive astrogliosis, measured by consistently high CSPG release, in 3D *in vitro* brain models is critical in order to maintain neuronal health and functional synapses^32,33^.

**Figure 1:**
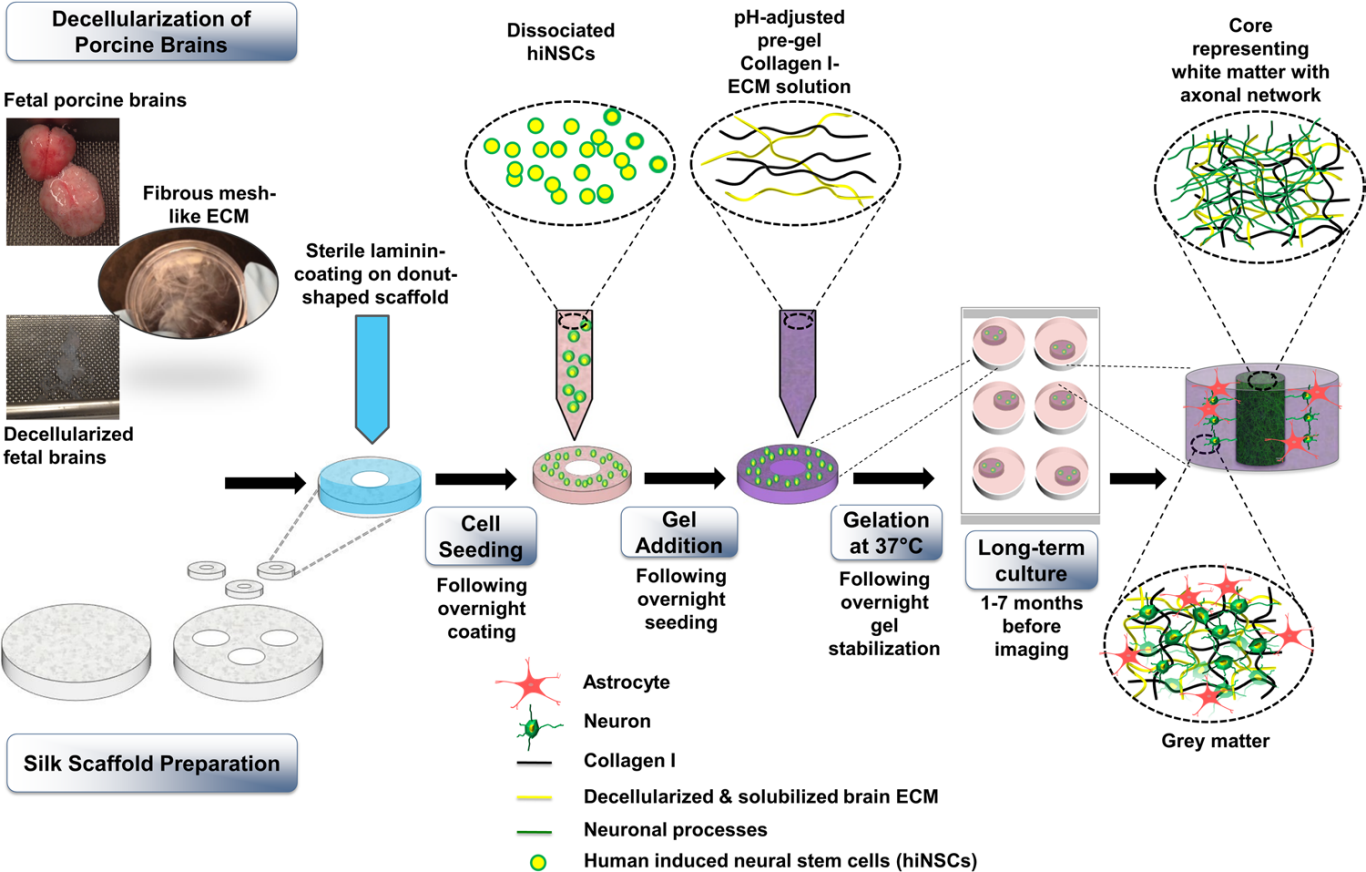
Culture of human induced neural stem cells in 3D *in vitro* bioengineered brain tissue constructs infused with decellularized brain ECM-collagen I hydrogel. The process starts with decellularization of porcine brains and silk scaffold preparation. Scaffolds are punched into 6 mm diameter constructs with a 2 mm diameter central hole. Laminin-coated scaffolds are seeded with dissociated human induced neural stem cells (hiNSCs). Decellularized ECM is mixed with collagen I and added to the scaffolds seeded with cells. The cell-seeded silk-scaffolds are flooded with media after complete gelation of ECM-collagen I. The center of the construct shows a dense axonal network, surrounded by the neuronal cell bodies and astrocytes. “Adapted with permission from Sood, Disha, *et al*. “Fetal brain extracellular matrix boosts neuronal network formation in 3D bioengineered model of cortical brain tissue.” *ACS biomaterials science & engineering* 2.1 (2015): 131–140. Copyright 2016 American Chemical Society.”

We hypothesized that the use of native brain-derived ECM for brain-relevant biochemical cues, in combination with a tissue engineered approach to design brain-specific tissue constructs would promote improved differentiation of stem cells into neurons and glia; as well as address the need for reproducibility, and tunability for compartmentalization/sampling. Many ECM proteins are conserved across species^34,35^, thus porcine brain-derived ECM was used towards the differentiation of hiNSCs. There are some differences expected, for instance, in the sulfation patterns of the GAG chains; but overall, porcine ECM is a good approximation for tissue-specific ECM involvement, as has been studied in the context of many different tissues^36–38^. In the current study, we investigated the effects of brain-derived ECM from two different developmental stages (fetal versus adult) on the differentiation of previously characterized hiNSCs^39^ into neurons and healthy astrocytes, when cultured within relevant environmental cues (biochemical factors and 3D topology) (Fig. 1). With the likelihood of epigenetic changes to be maintained in directly reprogrammed cells in comparison to iPSCs, as noted in previous studies^40,41^, we anticipate that hiNSCs-based models will better recapitulate neurodegenerative disease phenotypes. hiNSCs were cultured in bioengineered silk protein scaffold-based 3D tissue constructs infused with collagen type I (CLG1, previously shown to be compatible with brain cells^42^) hydrogels supplemented with native brain-derived ECM (fetal or adult).

**Figure 2:**
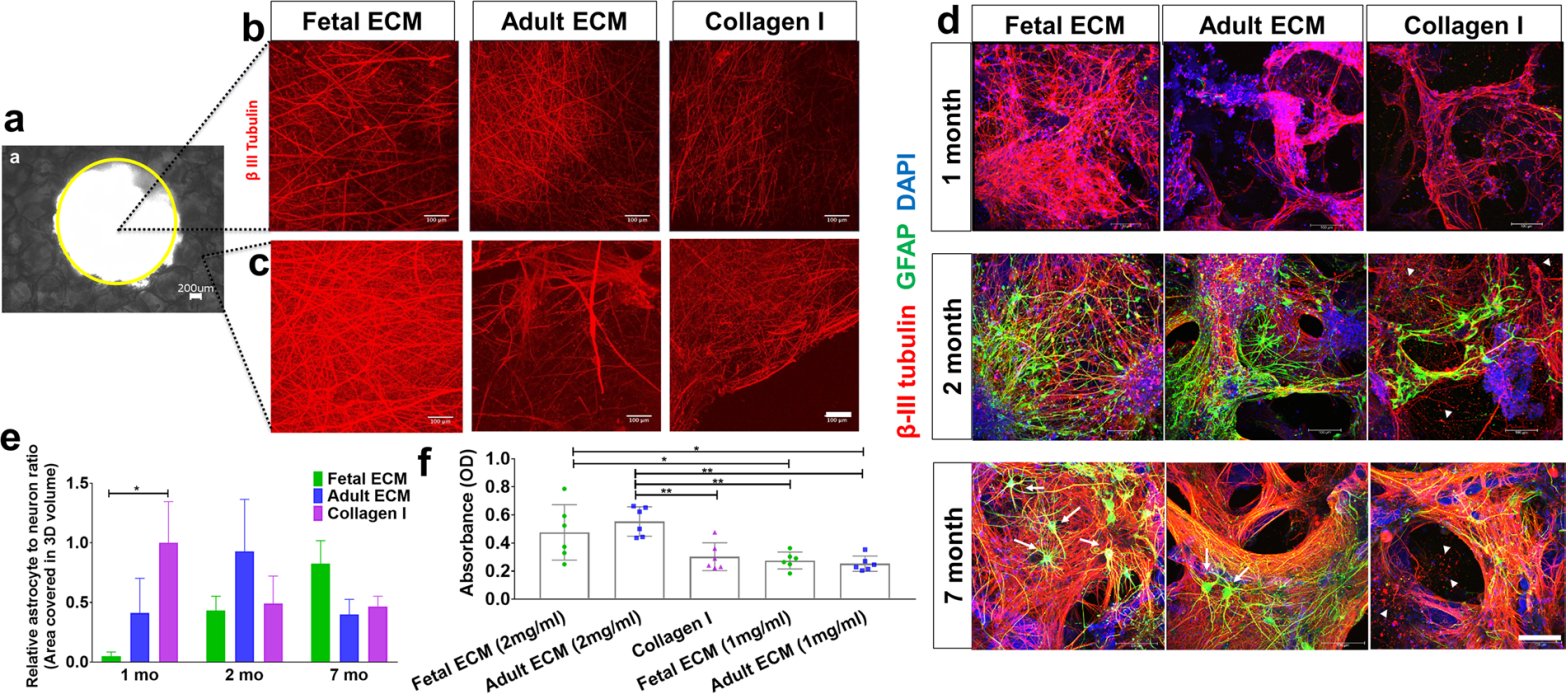
Extracellular matrix and time-dependent differentiation of human induced neural stem cells in 3D cultures. Human induced neural stem cells (hiNSCs) in silk scaffold-based 3D constructs infused with collagen I hydrogels supplemented with native porcine brain-derived ECM. (**a**) Brightfield image of silk scaffold with the middle circular window indicated by the yellow outline. (**b**) Growth of differentiating hiNSCs at 6 wk. shown by β-III tubulin staining for neurons within the middle hydrogel window of the 3D donut-shaped constructs. Max projection of z-stack. Scale bar 100 μm. (**c**) Growth of differentiating hiNSCs at 6 wk. shown by β-III tubulin staining for neurons within the ring portion of the 3D donut-shaped constructs. Max projection of z-stack. Scale bar 100 μm. (**d**) Growth and differentiation of hiNSCs at 1, 2 and 7 mo. shown by β-III tubulin staining for neurons (red) and GFAP staining for astrocytes (green) across different ECM conditions. Max projection of z-stack. Scale bar 100 μm. Arrows point to the star shaped astrocytes and arrow heads to the disintegrated axons and neuronal debris. (**e**) Astrocyte to neuron ratio calculated by dividing the total volume in 3D confocal stacks covered by astrocytes versus neurons post image processing. Mean ± SEM, One-way ANOVA with Dunnett’s post hoc test (Collagen I as control condition) at each time point on log transformed data, n = 3–6 individual scaffolds per condition. (**f**) Wst-1 viability assay at 2.5 mo. in 3D hiNSC cultures. One-way ANOVA with Tukey’s post hoc for multiple comparisons. *p < 0.0431, **p < 0.0071.

### Extracellular matrix and time-dependent differentiation of hiNSCs in 3D cultures

We tested whether the presence of brain-derived ECM cues accelerated the differentiation of hiNSCs into mature neurons and glia, particularly astrocytes. In the 3D bioengineered cultures, collagen type I was mainly used as a base matrix into which brain ECM was incorporated, as it was deemed suitable for long term culture and for neuronal growth after screening different commercially available matrices^43^. An increased density of axonal network from differentiating neurons was observed in fetal ECM-enriched constructs as shown by beta-III tubulin staining at 6 weeks, within both the axon rich central window (Fig. 2a,b) and the scaffold portion of the constructs (Fig. 2a,c).

Additionally, time-dependent increased differentiation of hiNSCs into neurons and astrocytes was observed based on immunostaining (Fig. 2d). The astrocyte population was more evident in 2-month cultures, closely following the differentiation of neurons. Star shaped astrocytes, suggestive of mature resting astrocytes, were only visible in the brain ECM-enriched constructs, particularly in 7-month cultures (Fig. 2d, marked by white arrows). The structural integrity of the neurons and astrocytes was maintained throughout the culture duration within the fetal brain ECM-enriched 3D brain tissue constructs (Fig. 2d, left column). In contrast, unhealthy neuronal morphologies, visible either as traces of disintegrated axons or as debris of clumped neuronal cell bodies, were present in the unsupplemented collagen type I constructs at all time points, and were particularly evident at 2 and 7 months (Fig. 2d, right column, marked by white arrow heads).

These qualitative observations were followed by the quantification of the volume covered by neurons and astrocytes within the 3D confocal stacks (n = 3–6 per condition). The volume covered by neurons was significantly (p = 0.034) greater in the fetal ECM constructs than adult ECM or unsupplemented collagen I at 1 month, while the astrocytic population was not evident (Fig. 2d,e, Supplementary Figure 1). A time-dependent increase in the ratio of astrocytes to neurons was confirmed in the fetal ECM constructs with an initial surge of astrocytes at 2 months (Fig. 2d,e). The inclusion of porcine brain-derived ECM had no toxicity as shown by wst-1 assay and lactate dehydrogenase (LDH) release profiles across all conditions (Fig. 2f, Supplementary Figure 2).

**Figure 3:**
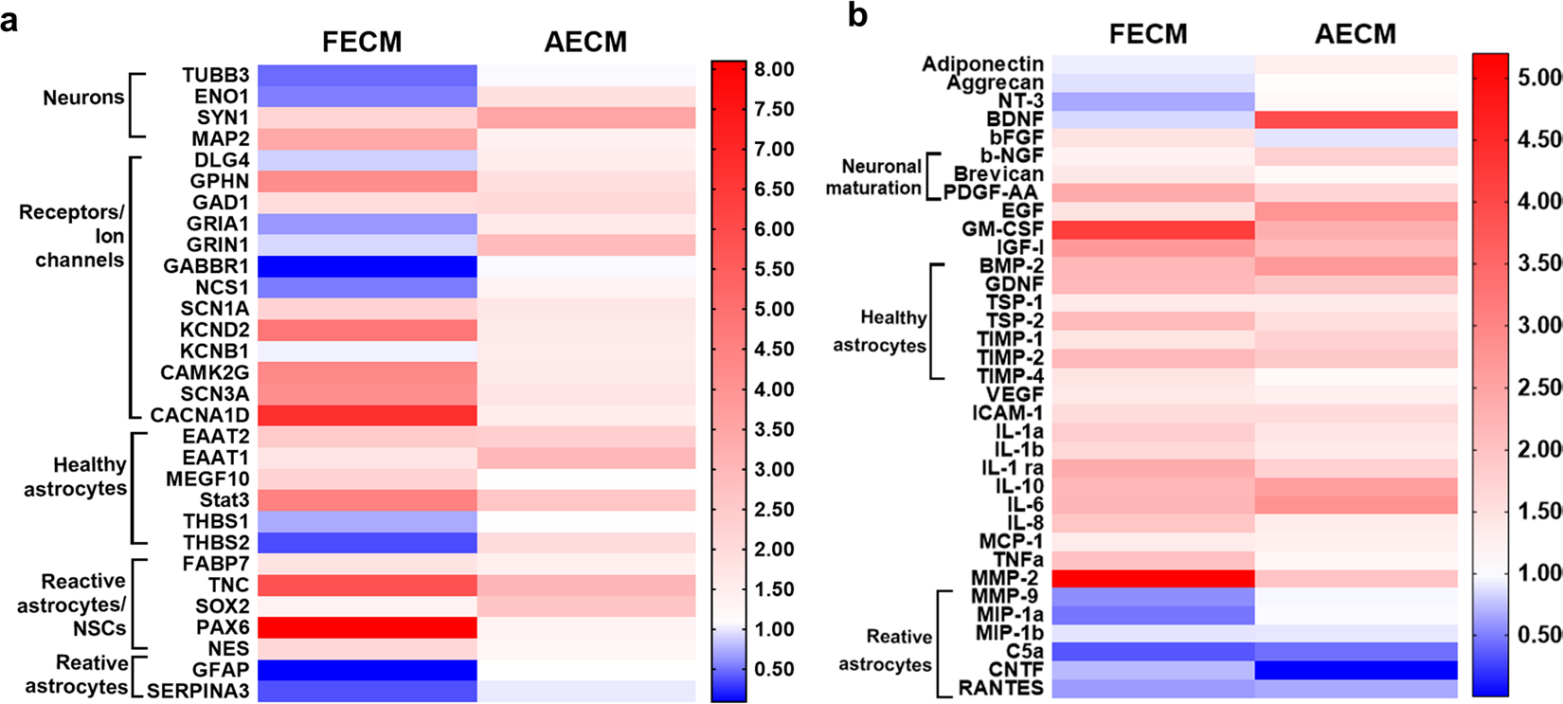
Gene expression and secretome changes in 3D bioengineered human induced neural stem cell cultures (hiNSCs) cultured in decellularized fetal or adult brain ECM. (**a**) Left and right panels indicate fold change in gene expression within fetal ECM and adult ECM-enriched constructs relative to collagen I condition, respectively. n = 3 pooled per condition at 1 month. (**b**) Cytokine release profile of differentiating hiNSCs in 3D bioengineered cultures at 1 month. Media was pooled from n = 7 samples per condition for the cytokine microarray. It can be noted from the heatmap scale that overall much more upregulation was observed than downregulation. Refer to Supplementary Tables 1–2 for the detailed list of genes and cytokines.

qPCR of 3D bioengineered hiNSC cultures was performed to determine the gene expression profiles of the differentiating cells (Fig. 3a). Genes corresponding to neurons, ion channels/receptors involved in calcium signaling, mature resting astrocytes, toxic reactive astrocytes, trophic reactive astrocytes, and neural stem cells were assessed. In 1-month cultures of fetal brain ECM-enriched tissue constructs, there was an upregulation of mature neuronal markers including synapsin 1 (SYN1) and microtubule associated protein 2 (MAP2), and mature heathy astrocytes including excitatory amino acid transporter 1 (EAAT1), excitatory amino acid transporter 2 (EAAT2)^10^, and multiple epidermal growth factor-like domain protein 10 (MEGF10)^44^. All of these markers were higher than in unsupplemented collagen I cultures of similar age (Fig. 3a). On the other hand, markers of toxic reactive astrocytes, such as Serpina3^45^, were downregulated in fetal brain ECM-enriched tissue constructs (Fig. 3a). Concurrent upregulation of many different voltage gated ion channels (sodium, potassium and calcium) was evident in brain-ECM supplemented constructs, particularly with fetal brain ECM (Fig. 3a).

Secretome profiling was used to characterize the cytokine release profile of differentiating hiNSCs. Soluble cytokines that are known to be important in astroglial differentiation (e.g., bone morphogenetic protein (BMP)^46^, glial derived neurotrophic factor (GDNF)^47^) and in generating/maintaining healthy neurons/synapses (e.g., Brevican^48^, PDGF-AA: platelet-derived growth factor AA^49^, b-NGF: beta nerve growth factor^50^, thrombospondins (TSPs)^51^), were released in relatively greater amounts in brain ECM-enriched constructs in comparison to unsupplemented collagen I (Fig. 3b). On the other hand, many cytokines associated with reactive astrocytes, including complement component 5a (C5a), Chemokine ligand 5 (RANTES)^52^ and MMP-9^53^, were higher in unsupplemented collagen I cultures (Fig. 3b). Thus, modulation of hiNSC differentiation into neurons and astrocytes was achieved using decellularized ECM derived from specific developmental stages, with fetal brain-derived ECM resulting in overall highest upregulation and release of neuron supporting factors.

### Chondroitin sulfate proteoglycan secretion as a marker of astrocyte maturity and reactive astrogliosis

**Figure 4:**
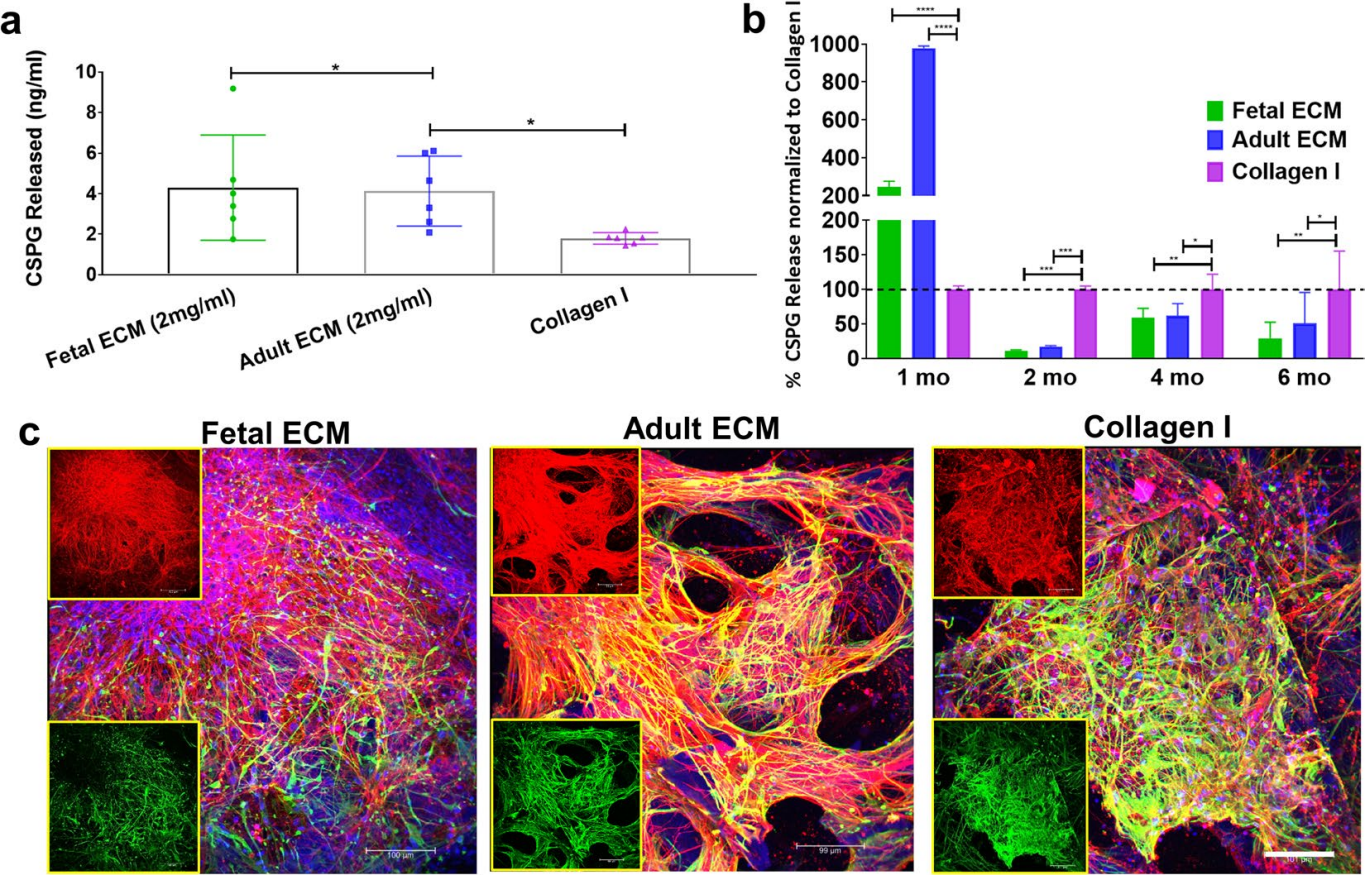
Chondroitin sulfate proteoglycans as a marker of astrocyte maturity or reactive astrogliosis. The amount of chondroitin sulfate proteoglycans (CSPGs) released in media by the cells within 3D constructs. (**a**) CSPGs released in media from collagen-based 3D constructs at 1 wk., expressed in ng/ml. Ordinary way ANOVA on log transformed data followed by Tukey’s posthoc test. (**b**) Percentage release of CSPGs in media from collagen-based 3D constructs at different time points. Ordinary two-way ANOVA with Dunnett’s post hoc test and Collagen I as control condition, n = 3–6. (**c**) Differentiating hiNSCs at 3 mo. shown by β-III Tubulin staining for neurons (red) and GFAP staining for astrocytes (green) across different ECM conditions. Insets show the red and green channels separately. Max projection of z-stack. Scale bar 100 μm. *p < 0.0407, **p < 0.0097, ***p < 0.0003, ****p < 0.0001.

During development, chondroitin sulfate proteoglycans (CSPGs) are transiently upregulated and produced largely by maturing neurons and astrocytes; however, during disease states reactive astrocytes exhibit sustained upregulation of CSPG expression/secretion^54^. Relative CSPG levels can thus be utilized as an indicator of astrogliosis^33^. CSPGs released in 1-week and 1-month hiNSC cultures were significantly higher in fetal brain ECM-enriched constructs compared to pure collagen type I hydrogels (*p = 0.0407, ****p < 0.0001) (Fig. 4a,b), as measured in an ELISA. There was a significantly lower (*p < 0.0407, **p < 0.0097, *** p < 0.0003) level of CSPG release in the brain ECM constructs at every time point post the initial month (Fig. 4b). At longer culture durations, unsupplemented collagen type I containing cultures consistently showed the highest levels of CSPG release (Fig. 4b). Additionally, the known morphological changes associated with reactive astrogliosis, including rapid proliferation and overlapping of cellular regions, was primarily seen in unsupplemented collagen type I matrices (Fig. 4c). Therefore, based on the CSPG release profiles and GFAP-stained cell morphology (Figs. 2d and 4), even the nominal presence of native brain-derived ECM supported the differentiation and maintenance of healthy astrocytes when grown long-term in 3D cultures (at least up to 7 months); as opposed to culturing in pure collagen type I matrix.

### Extracellular matrix dependent function observed in long-term 3D cultures

Calcium wave propagation in the developing brain has been implicated in the regulation of diverse cellular differentiation, through modulation of neurotransmitter expression, and axon and dendritic morphogenesis^55^. Differentiated neuronal and glial populations also exhibit robust and cell-specific calcium activity, including single cell spikes or network bursts^56^. For instance, developing neurons have been shown to have increased spontaneous burst activity during the formation of synapses and networks related to their stabilization^57,58^. Thus, the spatiotemporal patterns of calcium signaling in the 3D developing cultures were assessed to decipher the role of ECM in modulating spontaneously functional networks.

**Figure 5:**
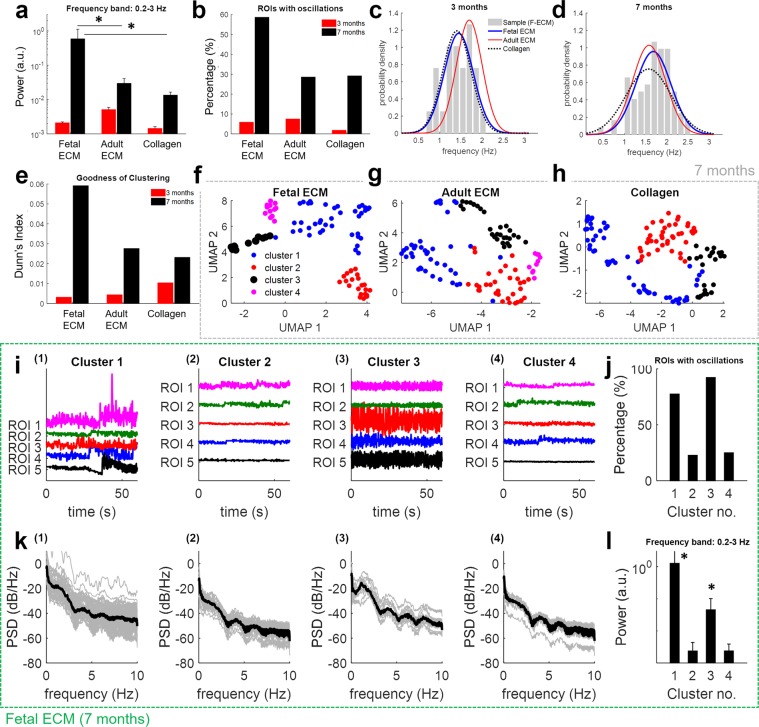
Effects of ECM on spontaneous calcium activity in 3D cultures of differentiating human neural stem cells. Panels a–h) summarize the results of the spectral analysis and cluster analysis of the change in fluorescence ΔF/F for ROIs identified at 3 months and 7 months from constructs with fetal ECM, adult ECM, and Collagen I. Panel a) reports the power in the frequency band [0.2, 3] Hz for Δ*F*/*F* signals at 3 months (red bars) and 7 months (black bars). Values are mean ± S.E.M. across *N* ROIs. Values at 3 months: *N* = 341 (fetal ECM), 294 (adult ECM), 277 (Collagen I) from *n* = 3 cultures. Values at 7 months: *N* = 87 (fetal ECM), 112 (adult ECM), 120 (Collagen I) from *n* = 3 cultures. Asterisks denote significant difference between groups at 3 months versus 7 months and between groups with different ECM constructs (two-way ANOVA with Tukey’s *post hoc* test, *P*-value *P* < 0.01). Panel b) reports the percentage of ROIs at 3 months (red bars) and 7 months (black bars) whose Δ*F*/*F* time series exhibit significant oscillations for different types of ECM constructs. Percentages are calculated over *N* ROIs, with *N* as in panel a). Panel c,d) report the probability distribution function (PDF) of the frequency *f* of signi*f*icant oscillations detected in ROIs from fetal ECM (blue lines), adult ECM (red lines), and Collagen I (dotted black lines) at 3 months (**c**) and 7 months (**d**). Each PDF is a Normal function fitted on the sample distribution of frequency *f*. Gray bars in (**c,d**) report the sample distribution of frequency *f* for ROIs *f*rom fetal ECM (F-ECM) constructs at 3 months and 7 months, respectively. Panel e) reports the Dunn’s index for the clustering performed on *N* ROIs at 3 months (red bars) and 7 months (black bars) from constructs with fetal ECM, adult ECM, and Collagen I. Values of *N* are as in panel a). Dunn’s index is computed on the UMAP components of the feature vectors. See *Methods* section for details. Panel f–h) report the UMAP plot of the ROIs from fetal ECM (**f**: *N* = 87), adult ECM (**g**: *N* = 112), and Collagen I (**h**: *N* = 120) constructs at 7 months colored by four clusters. Clustering was conducted separately for each construct. Panel i–l) recapitulate the behavior of spontaneous calcium activity from different clusters isolated at 7 months in constructs with fetal ECM. For cluster 1 through 4, Panel i) reports fluorescence signals Δ*F*/*F* from five sample ROIs from the cluster, while Panel k) reports the power spectrum density (PSD) of all ROIs in the cluster (gray lines, one line per ROI) and their median PSD (thick black line). Sub-panels (1), (2), (3), and (4) in (**i**–**k**) are for cluster 1, 2, 3, and 4, respectively. Number of ROIs per cluster are *M* = 40 (cluster 1), 22 (cluster 2), 13 (cluster 3), and 12 (cluster 4), respectively. Panel j) reports the percentage of ROIs in each cluster whose Δ*F*/*F* time series exhibit significant oscillations. Panel l) reports the power in the frequency band [0.2, 3] Hz for Δ*F*/*F* signals in each cluster. Values are mean ± S.E.M. across *M* ROIs, with *M* as in (**i**–**k**). Asterisks denote significant difference between clusters (one-way ANOVA with Tukey-Kramer *post hoc* test, *P*-value *P* < 0.01).

We first looked at the power spectrum density (PSD) of the relative change in fluorescence (signal Δ*F*/*F*, see Methods) from isolated regions of interest (ROI) in each culture and measured the total power in the band [0.2, 3] Hz. We found that the highest power values were achieved for ROIs observed in fetal ECM-enriched constructs at 7 months (Fig. 5a), and that such values were significantly (two-way ANOVA test, *p* < 0.01) higher than the values obtained with adult ECM and collagen I-enriched constructs, both at 3 months and 7 months. In contrast, the activity at 3 months was generally low, with no significant difference among the constructs.

We also tested whether ROIs had spectral peaks that were significantly higher (i.e., three times standard deviation) than the 1/f power law decay component of the PSD^59^ and used these peaks to detect periodic oscillations in the calcium waves. Figure 5b shows that approximately 60% of the ROIs collected at 7 months from fetal ECM-enriched constructs (51 out of 87 ROIs) had a significant spectral peak whereas only 30% of the ROIs collected from the other constructs (i.e., adult ECM: 32 out of 112; Collagen I: 35 out of 120) at the same time point had a significant peak. Furthermore, the number of ROIs with oscillations was lower at 3 months, with no significant difference among the constructs. The frequency *f* of the periodic oscillations associated with these spectral peaks was normally distributed between 0.5 Hz and 3 Hz, with no significant difference between types of ECM-enriched constructs or time point (Fig. 5c,d). Together, these results indicate sustained oscillatory activity across the cultures involving fetal ECM-enriched constructs was detectable at 7 months and was significantly higher than in other constructs. No significant oscillatory activity, instead, was noticed at 3 months.

We then investigated whether the enhanced activity detected in fetal ECM-enriched constructs was uniform across ROIs or rather driven by clusters of cells with highly correlated characteristics in the time-frequency domain. Therefore, we computed a total of 8 features per ROI (see Supplemental Methods) to uniquely characterize the fluorescence activity, and for each combination of time point and type of ECM-enriched construct, we clustered the available ROIs according to the degree of similarity between the correspondent features. The similarity was measured using the *L*_2_ (i.e., Euclidean) distance between feature vectors and the clustering was conducted using the Louvain modularity algorithm^60^, which optimizes the number of clusters and the assignment of ROIs to each cluster in an unsupervised way. The goodness-of-fit of the resultant partition was assessed by measuring the Dunn’s index (DI)^61^, with high DI values indicating well-separated clusters.

Fig. 5e demonstrates that the clustering procedure resulted in poorly separated clusters for ROIs at 3 months regardless of the type of ECM-enriched construct, whereas well-formed clusters emerged at 7 months. In addition, the separation between clusters was maximal for ROIs from the fetal ECM-enriched constructs, with no significant difference between adult ECM-enriched and Collagen I-enriched constructs. This is further noticeable in Fig. 5f–h, where the clusters detected for fetal ECM-, adult ECM-, and Collagen I-enriched constructs at 7 months were reported using the uniform manifold approximation and projection method (UMAP)^62^. See Supplementary Fig. 3 for the UMAP components of the clusters estimated at 3 months for fetal ECM-, adult ECM-, and Collagen I-enriched constructs.

Furthermore, we looked at the presence of significant oscillations in each cluster and we found that, for fetal ECM-enriched constructs at 7 months, the ROIs with significant oscillations were mostly concentrated in two out of four clusters (Fig. 5i, sub-panel (1) and (3)). ROIs in these clusters presented a common oscillatory activity, which resulted in high percentages of ROIs with significant spectral peaks (Fig. 5j: 31 out of 40 ROIs in cluster (1), 12 out of 13 in cluster (3)), similar PSD (Fig. 5k, sub-panel (1) and (3)), and high power values in the range 0.2–3-Hz (Fig. 5l). Vice versa, the remaining clusters aggregated ROIs with modest activity, low-intensity spectra (Fig. 5I,k, sub-panel (2) and (4)), and weak oscillations (only 5 out of 22 and 3 out 12 ROIs presented significant spectral peaks in cluster (2) and (4), respectively). Results for clusters detected in adult ECM- and Collagen I-enriched constructs at 7 months are reported in the Supplementary Figs. 4 and 5, respectively; indicate that at most one cluster included ROIs with significant peaks, even though the fraction of active ROIs per cluster was significantly lower than in case of fetal ECM-enriched constructs.

Together, these results indicate that fetal ECM-based constructs allowed neural populations with a higher fraction of active, spontaneously spiking neurons, and within each cluster, a more intensely coordinated physiological activity.

### Transduction of hiNSCs with a neuron-specific reporter and genetically encoded calcium sensor for cell-specific tracking

**Figure 6:**
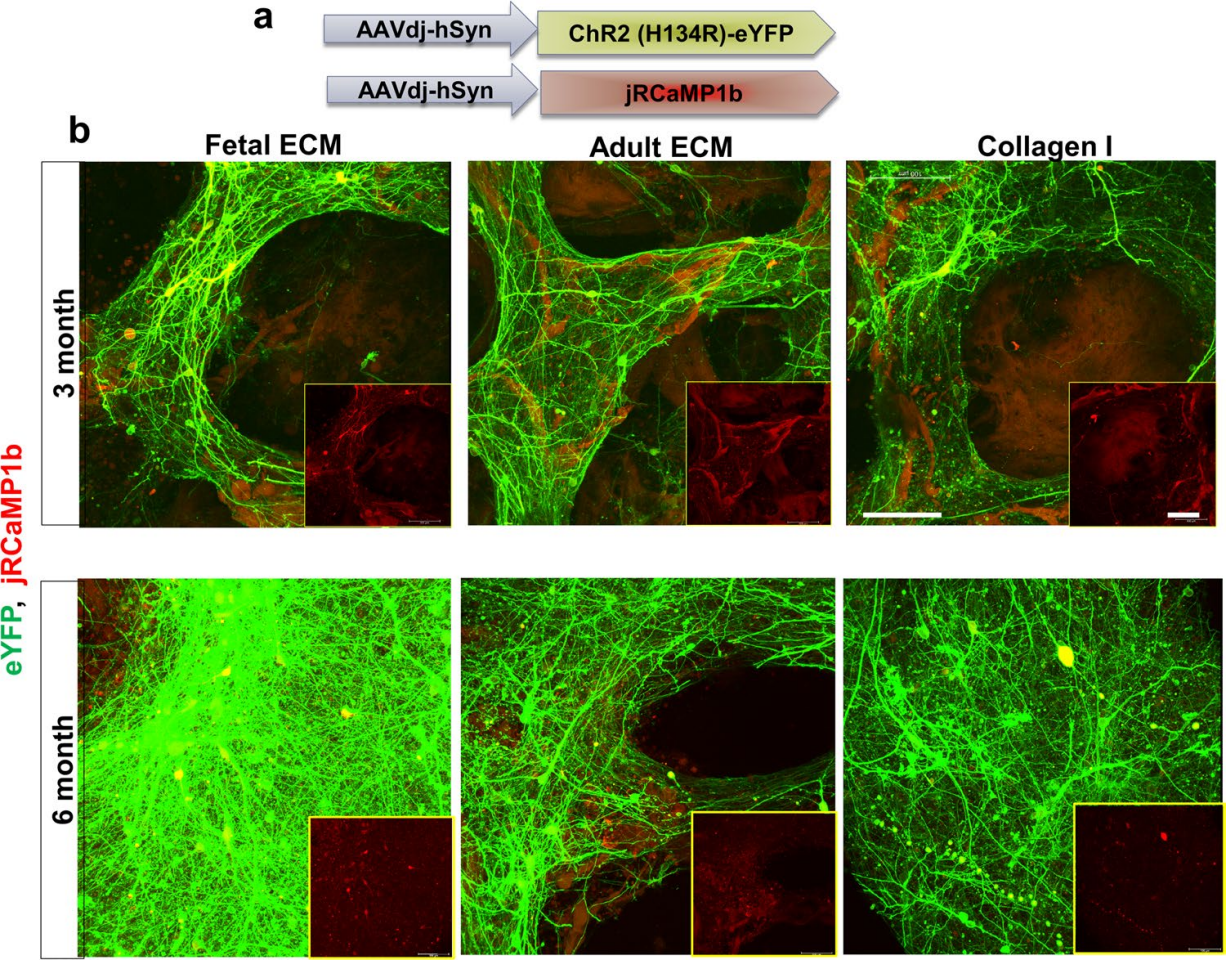
Transduction of differentiating human induced neural stem cells for tracking mature neurons. (**a**) High transduction efficiency virus, AAV-dj for expression of eYFP throughout the cell volume, a channelrhodopsin ChR2 (H134R) and calcium sensor jRCAMP1b under the synapsin promoter. (**b**) eYFP (green) and jRCAMP1b (red) expression at 3 and 6 mo. in mature neurons differentiated from hiNSCs in silk scaffold-based 3D constructs infused with collagen I hydrogels supplemented with native porcine brain-derived ECM. Insets show jRCAMP1b channel only. Max projection of Z-stack. Scale bar 100 μm.

To longitudinally track neuronal populations, differentiating hiNSCs were transduced with adeno-associated virus-dj (AAV-dj), a hybrid serotype with a higher transduction efficiency and infectivity *in vitro* in comparison to other wild type AAV serotypes^63^. The transduction virus enabled the expression of eYFP (yellow fluorescent protein) throughout the cell volume under the synapsin promoter, such that the arising mature neuronal populations could be tracked over time. Additionally, a genetically encoded calcium sensor, jRCaMP1b, was expressed in the differentiating hiNSCs under the synapsin promoter. The red-shifted calcium sensor, jRCaMP1b, was particularly chosen for several advantages; brighter/stable long-term expression over GCaMP6, imaging capability at greater depths with reduced photodamage, greater sensitivity and dynamic range before saturation^64^. This dual transduction enabled label-free tracking of mature neurons over time in the cultures, while simultaneously allowing for visualization of calcium levels (Fig. 6a). We confirmed the presence of mature neurons expressing eYFP and jRCaMP1b at both 3 and 6 months in the 3D bioengineered cultures across all ECM conditions (Fig. 6b upper and lower panels, respectively). Qualitatively, the neuronal networks were more intact and structurally robust in the fetal ECM-enriched constructs, with overall higher baseline calcium levels and increased network density at 6-months (Fig. 6b, lower panel). Thus, differentiating neuronal populations were successfully transduced with dual viral constructs to specifically track these populations.

### Composition analysis of decellularized brain extracellular matrix

**Figure 7:**
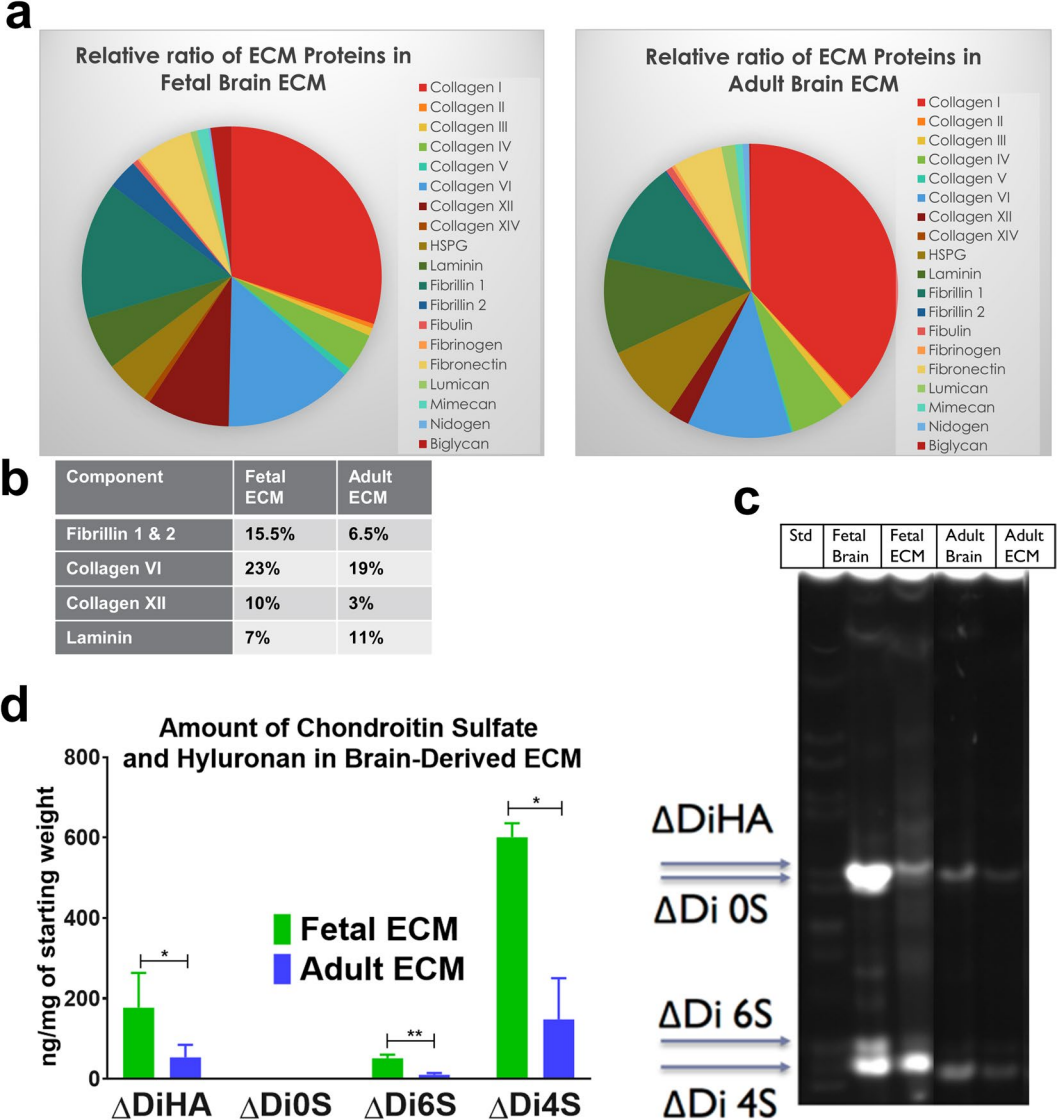
ECM composition analysis using LC/MS and fluorescence assisted carbohydrate electrophoresis. **(a**) Relative ratios of ECM proteins in fetal versus adult porcine brain decellularized matrix obtained through LC/MS. (**b**) Table indicating the relative percentages of a few select ECM components in decellularized brain matrix. (**c**) Characterization of decellularized fetal & adult brain ECM in comparison to fetal and adult whole brains by FACE. Chondroitin sulfate (CS) and hyaluronan (HA) bands in the fetal & adult porcine brain ECM. ΔDiHA: hyaluronan, ΔDi0S: non-sulfated CS, ΔDi6S: 6-sulfated CS, ΔDi4S: 4-sulfated CS. (**d**) Overall higher quantity of GAGs present in fetal brain ECM, as opposed to adult brain ECM. Unpaired two-tailed t-tests on log-transformed data between individual pairs, n = 3, *p < 0.0439, **p = 0.0040.

We sought to characterize the composition of fetal and adult brain matrices, which potentially contributed to the observed phenotypic changes, differentiation capacity and functionality upon culture of hiNSCs in these matrices. Through Liquid Chromatography-Mass Spectrometry (LC/MS), we confirmed that a complex composition of brain ECM was maintained post-decellularization. The components varied in relative amounts between fetal versus adult ECM, but included both fibrous proteins (up to 8 types of collagens, fibronectin, laminin), glycoproteins (nidogen, fibulin, fibronectin, fibrillins) and non-fibrous proteoglycans (heparin sulfate proteoglycans/HSPGs, biglycan, mimecan, lumican) (Fig. 7a). Fibrillins were higher in fetal brain-derived ECM (Fig. 7b). Furthermore, biglycans were enriched in the decellularized fetal brain ECM, when tested over multiple different extractions (Supplementary Fig. 6a).

Considering a significant loss of GAGs during sample processing for LC/MS, we resorted to a specialized technique, Fluorescence Assisted Carbohydrate Electrophoresis (FACE), for GAG compositional analysis of the decellularized brain ECM^65,66^. FACE analysis of whole brain tissue indicated a greater amount of GAGs present in fetal versus adult brain (Fig. 7c, Supplementary Figure 6b-d). This trend was maintained in the decellularized brain, with fetal ECM indicating significantly higher levels (p < 0.0439) of GAGs versus adult ECM, specifically 4-sulfated chondroitin sulfate (4S-CS), 6S-CS and HA (Fig. 7c,d). Additionally, HSPGs were present in higher amounts in fetal ECM (Supplementary Figure 6d).

## Discussion

There has been a growing interest in the use of *in vitro* 3D brain-like tissue systems for the study of neurological diseases using patient-derived stem cells^12,67–69^. Some of the limitations of these systems include inconsistent and slow co-differentiation of the cells towards neurons and supporting cells, such as astrocytes. Notably, Matrigel-based ECM hydrogels utilized for brain organoids, the current state of the art technology in the field, are poorly defined; this leads to variable differentiation effects such as inconsistencies in cortical layer formation and represented brain regions, including the presence of non-ectodermal identities^70^. Although, regionally specific brain ECM networks are reported to play major roles in generation of structural and functional diversity, little is known about how ECM is developmentally regulated in the brain^70,71^. To our knowledge, the differentiation effects of fetal versus adult brain ECM on human NSCs have not been characterized previously. Our previous study laid the foundation for the instructive role of decellularized fetal brain ECM in boosting primary mice neuronal culture^43^. The current study investigated the composition and role of developmental stage-sourced brain ECM for enhanced and functional maturation of human induced neural stem cells (hiNSCs) into a co-culture of healthy neurons and astrocytes.

We sought to investigate the effects of developmentally sourced brain ECM in 3D bioengineered tissue systems. Although silk is not present in brain tissue, we utilized silk as a scaffolding material for multiple reasons. The advantages of a silk scaffold-based model proposed here include structurally robust long term cultures without necrosis in the core, ease of handling and reproducibility, segregation of neuronal cell bodies and axons with ease of monitoring neural network formation over time, and increased surface area for cell attachment in 3D. We hypothesized that 3D brain constructs generated using brain-derived ECM would create a more physiologically relevant microenvironment conducive to growth and maturity of hiNSCs. We utilized hiNSCs directly reprogrammed from dermis-derived cells instead of induced pluripotent stem cells (iPSCs) because they differentiate rapidly and efficiently into both neurons and glia, without the need for lengthy protocols required for iPSC differentiation that can vary in efficiency^39^. Here, we report functional networks with enhanced maturation of hiNSCs, predominantly in fetal brain ECM-enriched cultures at 7 months. These functional results correlated with the upregulation of various ion channels in fetal ECM cultures.

Achieving a mature astrocyte population with temporal and physiological relevance that recapitulates the phenotype of healthy astrocytes *in vivo* is crucial. Greater differentiation of hiNSCs into healthy astrocytes was expected in the presence of native brain-derived ECM due to the neuroinductive biochemical cues, leading to the generation of a more representative 3D *in vitro* model. Indeed, we report reactive astrocyte morphology, and consistent upregulation of CSPGs in cultures lacking brain-derived ECM. This is potentially the result of reactive astrogliosis in the pure collagen I cultures at longer durations, because reactive astrocytes have been predominantly indicated for CSPG upregulation and sustained secretion in adult central nervous system^72,73^. For instance, reactive astrocyte-derived CSPG subtypes including versican V2, neurocan and phosphocan have been shown to hinder axonal growth in spinal cords of amyotropic lateral sclerosis (ALS) patients^74^. In healthy cultures, CSPGs are expected to be lowered and stabilized mainly in perineuronal nets (PNNs); this is following the initial surge and in relevance to brain development where maturing neurons/astrocytes transiently upregulate CSPGs^75^. This was the case for brain ECM-enriched cultures, which also indicated the presence of morphologically healthy mature astrocytes^76^. Additionally, we approximated the subtypes of differentiated astrocytes within brain ECM-enriched 3D bioengineered brain cultures via gene expression arrays and secretome profiling. Resting mature astrocytes identified by markers such as glutamate transporters (EAAT1, EAAT2) and phagocytic genes (MEGF10)^10^ rarely proliferate; but assume a reactive morphology and distinct markers upon activation either towards the toxic pro-inflammatory A1 or the trophic A2 subtypes^77^. The existence of these two polarized populations of reactive astrocytes has been postulated, similar to macrophage polarization to subtypes M1 and M2^56^. The pro-inflammatory A1 reactive astrocytes lose normal astrocytic functions such as neuronal outgrowth, synaptogenesis and phagocytosis, and instead contribute to neuronal death by glial scar formation and by releasing toxic factors^45,78^. The secretome profiling of the 2 subtypes have indicated preferentially higher release of thrombospondins from the A2 trophic astrocytes, which was noted to be the case for brain ECM containing cultures. Notably, the peptidase inhibitor serpina3a, which is postulated to be a specific marker of A1 reactive astrocytes^79,80^, was downregulated within fetal brain ECM cultures.

Additionally, our results indicated that differentiated neurons appeared early, while astrocytes arose with increasing time in culture. The initial surge in neuronal maturation within the fetal ECM-enriched constructs leveled off at later time points. These responses are in line with the known switch towards astroglial differentiation of a multipotent cell, which initially gives rise to neuronal precursors through changes in receptor expression^30^. Moreover, maintenance of a stable astrocyte-to-neuron ratio with relevance to known *in vivo* values (~1.4), is critical in *in vitro* brain tissue models, since this ratio is dynamic initially during development and known to increase in disease states^81^. We report an astrocyte to neuron ratio with *in vivo* relevance in the generated *in vitro* brain tissue models when supplemented with fetal brain-derived ECM.

Delineating the basis for the divergence of observed cellular responses through the biochemical analysis of the brain-derived ECM may have implications for generating 3D *in vitro* brain disease models that are more representative with sufficient maturity levels. For instance, we noted overall higher amounts of fibrillins (Fig. 7b) and biglycans in fetal ECM (Supplementary Figure 6a), which could present a potential mechanism for control of reactive astrogliosis and warrants further inquiry. Fibrillin 1 expression is developmentally regulated and it acts as a reservoir for transforming growth factor-β (TGF-β) in ECM. Downregulation of fibrillin 1 has been associated with increased TGF-β signaling^82^. TGF-β on the other hand is a known inducer of reactive astrogliosis^33^. We postulate that the fibrillins present in decellularized brain ECM could potentially harbor specific growth factors, such as TGF-β, and thus, control reactive astrogliosis in long-term 3D cultures enriched with brain ECM. Moreover, biglycan proteoglycans have been noted for their role in maintaining synaptic stability^83^. On the other hand, adult brain ECM was noted to have modestly high levels of biglycans in comparison to fetal ECM (Supplementary Figure 6a) and thus, likely had a correspondingly modest effect towards hiNSC differentiation and relevant gene expression. We further attribute the overall favorable effects of fetal brain ECM on the maturation of hiNSCs to the fact that this prenatal brain matrix allows for plasticity during development. Similarly, decellularized zebrafish brain, known for its remarkable plasticity and CNS regeneration capability, was recently shown to promote rat cortical neuronal viability and network formation in a scaffold-based culture system^84^.

It should be noted that decellularization methods always involve some loss of ECM components depending on the decellularization agents^85^. While some loss is expected, we retained a complex mixture of ECM components^43^ in order to start addressing the importance of native ECM matrix for effective differentiation of hiNSCs; the goal in the current study. Further fractionation and analysis of ECM components will be needed to decipher the role of specific components towards differentiation of NSCs, which can eventually inform the development of a controlled brain-relevant matrix. The scaffold-based 3D tissue system is well suited to undertake studies not only for deciphering the role of specific ECM components during brain development or disease, but also to examine the remodeling of supplemented ECM by the cultured cells. Additionally, the deposition of cell-derived ECM will need to be examined as was recently shown in an organoid culture system^86^.

Finally, through transduction of differentiating neurons with a construct encoding a fusion protein (ChR2-eYFP) under the synapsin promoter and a genetically encoded calcium sensor (GECI), we demonstrated that this system is amenable to live tracking as well as all optical interrogation of cells over long-term cultures. Similar approaches could be used to place an opsin and GECI under a healthy astrocyte promoter, with distinct spectral profiles from those placed under the neuronal promoter synapsin; which could enable studies of cell-cell interactions towards the generation of network patterns and dissect cell-specific signaling. Moreover, the resulting scaffold-based brain tissue model with concomitant presence of neurons and astrocytes is highly tunable from a structure-morphological perspective. This feature enables cell body/axonal segregation and presents potential for spatially controlled introduction of morphogens or other cell types in future iterations, such that their roles in neurodegenerative diseases can be investigated.

## Conclusions

First, we report healthy mature astrocyte morphology supported by relevant gene expression/cytokine release, and downregulation of CSPGs in cultures supplemented with brain-derived ECM. Our results indicate that differentiated neurons appeared early, closely followed by astrocytes. Such systems would be useful to investigate neurodegenerative disorders, many of which have implicated reactive astrogliosis as a cause or consequence. Next, the combined functionality of cells differentiated from hiNSCs in the 3D bioengineered tissue model, revealed greater overall spontaneous activity at 7-month versus 3-month cultures (Supplementary videos 1–6). Clear differences were observed across ECM conditions, including more active clusters overall, increased oscillatory activity, highest concurrent upregulation of voltage gated ion channels and downregulation of markers of toxic reactive astrocytes in the fetal ECM-enriched constructs. Furthermore, potential for live tracking of differentiating neurons in long-term 3D cultures was shown using genetically encoded biosensors.

This is the first study to examine the composition of decellularized brain ECM from different developmental stages, specifically glycosaminoglycans (GAGs), and to identify native stimulatory cues relevant for functional maturation of hiNSCs over long term in 3D bioengineered brain constructs supplemented with decellularized fetal and adult porcine brain ECMs. Moreover, the combination of the proposed measurements of neurons and/or astrocytes with functional optogenetic interrogation in future iterations holds the potential to understand the interactions between cell types in healthy versus diseased states and to help unravel cell-matrix crosstalk. Altogether, the knowledge gained has the potential to enable the development of brain-specific biomaterials for generating physiologically-relevant 3D *in vitro* brain models.

## Materials and Methods

### 3D bioengineered brain model with hiNSCs

Assembly of the bioengineered cortical tissue was performed as previously described with further optimization^87^. Briefly, porous silk 3D scaffolds were coated with 0.25 mg/mL laminin (Sigma-Aldrich) either overnight at 4 °C or for 2 h at 37 °C. The scaffolds were incubated in media at 37 °C for at least 30 mins to equilibrate the scaffolds for cell seeding. hiNSCs were obtained from dermis-derived human fibroblasts through direct reprogramming, as previously described^39^. The IRB approval for the cell source was obtained for a previous study that generated this human induced neural stem (hiNSC) cell line. Originally, the human neonatal foreskin fibroblasts (HFFs) were a gift from Dr. Jonathan Garlick, Tufts University^39^. Expanding hiNSCs were lifted off mouse embryonic fibroblasts (MEFs) using TrypLE Select and centrifuged at 3,000 rpm for 1.5 mins. The cell pellet was resuspended in hiNSC expansion media consisting of KnockOut Serum Replacement DMEM, GlutaMax, KnockOut SR, Antibiotic-Antimycotic and 2-mercapto, bFGF Basic. The resuspended cell solution was vortexed and passed through a 40 µm filter to achieve single cell suspension. The resulting single cells obtained from hiNSC colonies were seeded on the 3D ring-shaped silk scaffolds at a concentration of 0.5–1 million in 100 µl volume per scaffold. After overnight incubation of 100 µl hiNSC concentrated cell suspension per scaffold in a 96-well plate to maximize cell attachment to the laminin coated silk, the unattached cells were washed away with the hiNSC expansion media. Next, the hiNSC cell-seeded scaffolds were infused with 3 mg/mL rat tail collagen type I (Corning), a commonly used matrix or with collagen-native brain ECM composite hydrogels.

For the generation of ECM-collagen I hydrogels, porcine brain ECM from different developmental stages were obtained via a previously developed decellularization process^43^. Lyophilized ECM was solubilized with 1 mg/mL pepsin from porcine gastric mucosa in 0.1 N hydrochloric acid. The solubilization time for fetal and adult ECM at room temperature was approximately 16 and 24 h, respectively. Once solubilized, the ECM was mixed with hiNSC differentiation media (Neurobasal media supplemented with 1% B27, 1% glutamax and 1% anti-anti) at 1:1 ratio and neutralized using 1 M NaOH. The neutralized ECM solution was mixed with 3 mg/mL rat tail collagen type I for a final ECM concentration of 1,000 μg/ml or 2000 μg/ml and the gelation process started using NaOH. The ECM-collagen solution was kept on ice until complete gelation was required and was used within 2 h of preparation. After introduction within the cell-seeded scaffolds, the gelation was completed in 30 mins at 37 °C, following which more hiNSC differentiation media was added to each well with the constructs. The next day, each of the ECM containing cell-seeded 3D constructs was moved to a larger well of a 24-well plate with sufficient media. The cultures were tested for mycoplasma using the Mycoalert mycoplasma detection kit from Lonza and were mycoplasma free.

### Immunostaining and quantification of area covered by neurons versus astrocytes

At different time points (1, 2, 7 months) in 3D cultures, cells were evaluated for neuronal network density and differentiation into neurons and astroglial cells with immunostaining. The samples were fixed at different time points with 4% paraformaldehyde (PFA) solution in PBS. Fixation time was 20–30 mins for the 3D constructs. The cells were stained with beta-III tubulin and GFAP (Sigma-Aldrich) as markers for neurons and astrocytes, respectively. Primary antibody incubations were performed at 4 °C overnight, while the secondary antibody incubations were carried out at room temperature for 2 h. The volume covered in 3D stacks was measured using a custom code generated in MATLAB. Briefly, the 3D stacks corresponding to either beta-III tubulin or GFAP were binarized, such that there are only two possible values corresponding to each pixel (black or white). The volume covered was represented as the total number of positive pixels (black) divided by the total pixels (corresponding to either black or white pixels) of all the planes in the corresponding z-stack.

### Calcium imaging, spectral analysis and cluster analysis

Differentiated cell functionality was determined using live calcium imaging at 3 and 7 months in culture. Cells seeded on 3D scaffolds were immersed in extracellular solution: NaCl 140 mM, KCl 2.8 mM, CaCl_2_ 2 mM, MgCl_2_ 2 mM, HEPES 10 mM, glucose 10 mM, pH 7.4. Fluo-4 calcium sensitive dye was mixed 1:1 with 20% Pluronic F127. Next, Fluo-4 was diluted to a final concentration 1 μM in the extracellular buffer prewarmed to 37 °C. The Fluo-4 1 μM solution was applied on the scaffolds and incubated at 37 °C for 1 h. Upon incubation, the constructs were washed with the extracellular buffer to remove excess dye. The constructs were imaged using the Keyence BZ-X700. The images were taken with following setup: 15 ms exposure, 50 ms frame frequency, 512 × 512 pixels, 4 × 4 binning, 1200 frames/min at 37 °C. Images were processed offline using the NIH ImageJ software suite.

Regions of interest (ROIs) were extracted automatically from a series of calcium images over time, following a two-step approach. First, the variance of the brightness of each pixel through time was computed, allowing for the generation of a heatmap. The heatmap was convolved with a 2-D Gaussian kernel with a standard deviation computed from the resolution and magnification of the images, to ensure continuity and to reduce noise. Next, the local maxima of the filtered heatmap were found and used as seed points to isolate discrete regions, each representing an ROI. Finally, fluorescence intensity time traces were plotted on the center of mass of the discrete regions (single pixel data).

For each ROI, the relative change in fluorescence was calculated as Δ*F*/*F* = (*F* − *F*_0_)/*F*_0_, where *F* is the fluorescence time series and *F*_0_ is the baseline of *F* estimated from a ± 15 s sliding window. Due to the sparse activity, *F*_0_ was calculated as the average of all values below the 80^th^ percentile in *F*. Finally, a second order Savitzky-Golay filter was applied to the Δ*F*/*F* signal to remove noise while preserving the signal frequency span.

For each time point (i.e., 3 and 7 months) and type of construct (i.e., fetal ECM, adult ECM, or Collagen I), the ROIs isolated in *n* = 3 cultures were collected and a feature-based cluster analysis of the Δ*F*/*F* signals from these ROIs was conducted as in Tang-Schomer *et al*.^88^. Briefly, each Δ*F*/*F* was processed to compute a feature vector consisting of 8 distinct features (see definition in *Supplemental Methods*), which collectively provide a time-frequency characterization that is unique to the Δ*F*/*F* signal. Then, the ROIs were envisioned as nodes in a graph, and the link between any pair of nodes (*i*, *j*) in the graph was weighted by the reciprocal of the *L*_2_ distance between the feature vectors associated to ROI *i* and *j*, respectively. In this way, ROIs that had similar feature vectors (i.e., small distance) were strongly connected whereas ROIs that had largely different feature vectors (i.e., high distance) were weakly connected. Collectively, the weights associated to the links in the graph form the connectivity matrix *C* of the graph, which is a square matrix that uniquely defines the graph’s structure^89^. Finally, the Louvain algorithm for community detection^60^ was applied to *C*, and ROIs were clustered together if they were assigned to the same community. The Louvain algorithm determines the number of communities in an unsupervised way and assigns the ROIs to communities to maximize the modularity index (MI)^60^, which is a value between −1 and +1. A MI close to −1 indicates poor separation between communities, while a value close to +1 indicates that each cluster includes highly related ROIs while ROIs from different clusters are significantly different. Because the Louvain algorithm is a locally greedy optimization procedure, we repeated the community detection a total of 100 optimizations and a consensus partition method was implemented as in (Lancichinetti & Fortunato)^90^ to obtain a consistent cluster partition for each culture. For sake of visualization in Fig. 5f–h, the feature vectors in each cluster were represented using the uniform manifold approximation and projection method^62^.

We further investigated the presence of significant oscillations in the signals Δ*F*/*F* by performing a spectral analysis corrected for aperiodic 1/f power law decay as proposed by Haller *et al*.^59^. Briefly, the power spectrum density (PSD) was computed for every time series Δ*F*/*F* using the Welch’s method (window size: 8 s; overlap: 2 s). Then, the PSD (in log-10 scale) was detrended to remove the aperiodic 1/f power law decay component (i.e., a line between 0.2 Hz and 8 Hz), and peaks in the detrended PSD signal were selected if they were higher than three times the standard deviation of the detrended PSD. Finally, all selected peaks from every ROI across all types of construct (i.e., fetal ECM, adult ECM, and Collagen I), aging point (i.e., 3 months and 7 months), and culture (i.e., *n* = 3 per type of construct and aging point) were gathered to estimate the sample distribution of the spectral peak values. Peaks in the top 75 percentile were considered as a proxy for oscillatory patterns in the spontaneous calcium activity of cultures, and the frequencies *f* of these peaks were used to estimate the periodicity of the oscillations. Computations were conducted in MATLAB 2018b, The MathWorks, Inc., Natick, MA (USA) and Python 3.7 (packages fooof-py^59^ and umap-learn^62^).

### Transduction of hiNSCs with viral constructs

The starting viral titer of AAVdj-hSyn-jRCaMP1b and AAVdj-hSyn-hChR2(H134R)-eYFP (Stanford University Virus Core) was 1.78 × 10^13^ and 3.62 × 10^13^, respectively. The virus was diluted at 1:1000 dilution in cell culture media for differentiating hiNSCs. Each cell-seeded scaffold was incubated in 400 µl media containing virus and exposure to the virus was maximized by adding additional media in the same well for an entire week. One-week post-infection, virus-containing media was replaced with fresh media. Virally transduced tissue constructs were imaged with a multiphoton confocal microscope (TCS SP8, Leica) equipped with a Ti-Sapphire laser. During imaging, each sample was placed in a well of a glass bottomed (No. 1.5 coverslip) 24-well plate. The imaging chamber was maintained at 37 °C and humidified along with a continuous supply of 5% CO_2_.

### Analysis of brain-derived ECM

Lyophilized ECM samples were weighed and solubilized at 5 mg/ml in 0.1% sodium dodecyl sulfate (SDS) in PBS containing 5 M urea, 2 M thiourea and 50 mM Dithiothreitol (DTT). The samples were solubilized for ~24 hrs at 4 °C with gentle stirring. Following this step, the solubilized ECM samples were acetone precipitated for 2 hrs at −20 °C^91^. The obtained pellets following removal of the supernatant were sent for liquid chromatography tandem mass spectrometry (LC-MS/MS) at the Beth Israel Deaconess Medical Center Mass Spectrometry Core facility. The resulting spectral count data was scanned to find the most abundant ECM proteins (n = 2 per ECM condition was analyzed).

Additionally, Fluorophore-assisted carbohydrate electrophoresis (FACE) was performed on lyophilized adult and fetal porcine brain ECM samples, using a previously developed protocol for glycosaminoglycan (GAG) analysis^92^. Briefly, the dry weight of the ECM samples was measured, followed by digestion in 1 mg/ml proteinase K at 60 °C for 2 h. Hyaluronan and chondroitin sulfate present within the samples were digested using the following enzymes: chondroitinase ABC (Seikagaku 25 mU/ml) and hyaluronidase SD (Seikagaku 2.5 mU/ml). Following ethanol precipitation and proteinase K heat inactivation, the ECM samples within the resulting pellet were incubated in the enzymes at 37 °C overnight. A second ethanol precipitation step was performed, and the resulting hyaluronan/CS glycans were retained within the supernatants after centrifugation. The glycans were labeled with the fluorophore 2-amino-acridone (AMAC) by incubation at 37 °C for 18 h. Finally, the labeled samples and standard disaccharides were loaded onto gels at lower volumes of 2–5 µl as opposed to ~30 µl in protein gels, to aid in band resolving. Electrophoresis was performed at a constant 500 V for 1 h 15 mins and FACE imaging was accomplished using a UVP Chemi-DocIt2 515 integrated system. Band quantifications were performed in ImageJ. To calculate the concentration of disaccharides in ECM samples, sample band intensity was divided by the intensity of the respective known standard. Relative intensities of disaccharide bands across samples were normalized by the starting dry weight.

### Statistics

Statistical analysis was performed using GraphPad Prism 8 software (GraphPad, CA, USA). All data are expressed as mean ± SD with sample sizes of *n* ≥ 3, unless stated otherwise. The analysis methods utilized included ordinary one-way ANOVA, followed by Tukey’s *post hoc* or Dunnet’s *post hoc* test when assigning unsupplemented hydrogels as the control condition to determine the statistically significant differences for multiple comparisons, and unpaired two-tailed t-test for comparison of two groups, unless stated otherwise. In the spectral analysis reported in Fig. 5a–d, a two-way ANOVA analysis was used with Tukey’s *post hoc* test, where the time point (i.e., 3 months or 7 months) and the type of ECM construct (i.e., fetal ECM, adult ECM, or Collagen I) were the independent variables. In this latter analysis, the level of significance was determined at *P* < 0.01. Samples were chosen randomly for the different experiments to account for any potential unavoidable biological variability. This included randomization post cell seeding within the scaffold and before allocation into different groups for ECM hydrogel addition. For analysis of calcium imaging the investigator was blinded to the different groups.

## Supplementary Information

Supplementary Information

Supplementary Video 1

Supplementary Video 2

Supplementary Video 3

Supplementary Video 4

Supplementary Video 5

Supplementary Video 6

## Data Availability

All data is included within the manuscript and supplementary sections. A master source data file has been provided for Figs. 2–4, 6 and Supplementary Figs. 1 and 6a. Source data and Matlab codes used to generate Fig. 5 and Supplementary Figs. 3–5 are provided in a separate zip folder labeled- CalciumImaging_Analysis_Code_Files_DS-DK.
