# Supplementary material for: Functional maturation of human neural stem cells in a 3D bioengineered brain model enriched with fetal brain-derived matrix
Source: Sci Rep. 2019 Nov 29;9:17874. doi: 10.1038/s41598-019-54248-1 (PMC6884597; doi:10.1038/s41598-019-54248-1)
Supplement: Supplementary file 1 — Supplementary Information [file 41598_2019_54248_MOESM1_ESM.pdf]

Functional maturation of human neural stem cells in a 3D bioengineered brain model enriched with fetal brain-derived matrix

Disha Sood[1], Dana M. Cairns[1], Jayanth M. Dabbi[1], Charu Ramakrishnan[2], Karl Deisseroth[2], Lauren D. Black III[1], Sabato Santaniello[3], David L. Kaplan[1*]

Supplementary Methods

Viability Assay

Cell proliferation reagent WST-1 assay (Sigma-Aldrich) was performed at end time points before freezing the samples for PCR, according to the instructions provided by the manufacturer, to assess overall cell viability across different ECM conditions. Briefly, the samples were incubated for 1 h with WST-1 reagent diluted 1:10 (v:v) in culture medium, followed by a reading of the medium absorbance with plate reader (Molecular Devices) at 450 and 600 nm as the reference wavelength. Fresh medium was used as a baseline control and its average absorbance was subtracted from the value of the samples.

Lactate Dehydrogenase Assay

Lactate dehydrogenase (LDH) enzyme released into media by the ruptured cells, was used as a measure of cell death at different time points during the 3D culture without having to sacrifice the samples. LDH assay was performed according to the manufacturer instructions (Sigma-Aldrich). Briefly, culture medium was mixed with the assay reagents in a 1:2 ratio. Following 30 mins incubation at room temperature, the reaction was stopped by addition of 1N HCl. The absorbance readings were measured at 490 nm and 690 nm as the reference wavelengths. Fresh medium without any construct was used as a baseline control and its average was subtracted from the sample values.

CSPG Release ELISA

CSPGs released by the differentiating hiNSCs in media were measured using an ELISA based assay. Media samples from the 3D constructs were incubated overnight at 4°C in a 96-well immuno plate (Thermo Fischer Scientific). Alongside the sample media incubation, chicken extracellular CSPGs (Millipore) were used over a range of serial dilutions for the generation of standard curves. Following washes with PBS-tween, monoclonal anti-chondroitin sulfate antibody produced in mouse/clone CS-56, ascites fluid (Sigma) was added for overnight incubation at 4°C. After the next round of washes, HRP conjugated goat anti-mouse secondary antibody (Abcam) was incubated at room temperature for 2 h. TMB (3,3',5,5'-tetramethylbenzidine) 1-C Substrate (Fisher Scientific) was introduced following the last round of washes with PBS-Tween. Finally, after the color developed for 10 mins at room temperature, the reaction was stopped with 1N HCl. The absorbance readings were

measured at 450 nm wavelength and the fresh media readings were subtracted from the sample readings. The standard curve was utilized for calculating the quantities of CSPGs released in the different conditions and reported in pg/ml.

qRT-PCR

Samples were flash frozen in liquid nitrogen and stored in -80oC in individual Eppendorf tubes until RNA extraction was performed. All samples were placed on dry ice during extraction, sequentially disrupted using a liquid nitrogen chilled bio-pulverizer. Between each sample, the pulverizer was wiped with 70% ethanol to remove remnants of the previous sample, and between each sample set (different tumor types), all the tools were cleaned with RNAzap. Lysis buffer was immediately added to the powdered frozen sample and placed on ice. Once all the samples were in lysis buffer on ice, a 22 gauge needle and syringe was used for sample homogenization one by one using a fresh needle and syringe every time. All the samples were spun down to remove undigested material (mainly silk scaffold) and the supernatant was transferred to clean RNAse free-Eppendorfs. Following this, the SurePrep All Prep kit (Fisher Scientific) protocol was followed until RNA was eluted from the columns. RNA concentrations were measured using nanodrop 2000 (Thermo Fisher Scientific). RT2 First Strand Kit with an incorporated gDNA removal step with buffer GE (Qiagen) was utilized for cDNA synthesis from the eluted RNA. cDNA samples were mixed with RT^2 SYBR Green Fluor qPCR Mastermix and added to the Qiagen Custom RT2 PCR Array (including a housekeeping gene, genomic DNA control and RT control). PCR was run on BioRad CFX96. Table 1 lists the genes that were tested. The primers for all the genes profiled in qPCR were bought from Qiagen. These sequences were pre-designed and validated by Qiagen.

Cytokine Arrays

Multiplex Quantibody cytokine arrays (RayBiotech) were used to semi-quantitatively compare cytokines (Table 2) released by differentiating hiNSCs cultured in the 3D bioengineered brain model with different ECM components. Small volumes of control media samples/cell culture supernatants (50µl) from the 3D constructs were incubated in the capture antibody spotted glass slides or the membranes, along with the standards provided that corresponded to known concentrations of the targets for the Quantibody arrays. This overnight incubation was followed by another overnight step at 4°C, involving the biotinylated detection antibody cocktail. Next, streptavidin-conjugated fluorophore or HRP-streptavidin was added for 1 h at room temperature. Finally, the slide

was disassembled from the removable gasket, dried and scanned using a fluorescence microarray laser scanner (Ray Biotech). Protein expression profiles of the differentiating hiNSCs across the varying ECM conditions were quantified using the Q-analyzer software (Ray Biotech). Table 2 lists the cytokines that were tested.

Calcium Imaging Cluster Analysis

Each Δ*F*/*F* was processed to compute the following features, which collectively provide a time-frequency characterization that is unique to the Δ*F*/*F* signal:

1) Line-length: $f_1 = \frac{1}{N}\sum_{k=1}^{N-1} |\Delta F / F(k+1) - \Delta F / F(k)|$;

2) Standard Deviation: $f_2 = \frac{1}{N}\sqrt{\sum_{k=1}^{N}\left(\Delta F / F(k) - \mu\right)^2}$;

3) Entropy: $f_3 = -\sum_n p_n \log_2 p_n$;

4) Spectral peak: $f_4 = \arg\max_{\omega} P_{\Delta F/F}(\omega)$;

5) Spectral centroid: $f_5 = \frac{\int \omega P_{\Delta F/F}(\omega) d\omega}{\int P_{\Delta F/F}(\omega) d\omega}$;

6) Energy: $f_6 = \sum_{k=1}^{N}\left(\Delta F / F(k)\right)^2$;

7) Global/local peak ratio: $f_7 = \frac{g^*}{\sum_{g_i \in G,\, g_i < g^*} \frac{g_i}{J-1}}$,

where Δ*F*/*F*(*k*) is the *k*-th sample in the Δ*F*/*F* time series, *N* is the total number of samples in Δ*F*/*F*, *μ* is the average value of Δ*F*/*F*, and $P_{\Delta F/F}(\omega)$ is the power spectrum density of the Δ*F*/*F* signal at frequency $0 \leq \omega \leq F_s/2$, where F_s is the number of frames per second. To estimate the entropy (3), the sample probability function of the Δ*F*/*F* intensity values is computed and the correspondent sample probability values p_n are used. The spectral peak (4), instead, is the frequency *ω* of the maximum power spectrum density value $P_{\Delta F/F}(\omega)$. Finally, the peak ratio (7) is estimated by computing all the local maxima (i.e., peaks) $G = [g_1, g_2, g_3, \ldots, g_J]$ of the Δ*F*/*F* signal and the absolute maximum (i.e., global peak) g^* among the peaks in *G*. In addition to features (1)-(7), the entropy of the squared-normalized Teager Energy vector was computed. Briefly, the Teager Energy series was computed:

$$T(k)=\begin{cases}\Delta F/F(k)-\Delta F/F(k-1)\times\Delta F/F(k+1) & k=2,3,...,N-1\\ 0 & otherwise\end{cases}$$

and the sample entropy is computed as:

8) Teager Energy entropy: $f_8=-\sum_{k=1}^{N}S(k)\log_2 S(k)$,

where *S*(*k*) is the squared and normalized version of *T*(*k*), i.e., $S(k)=T^2(k)/\sum_j T^2(j)$.

Supplementary Figures

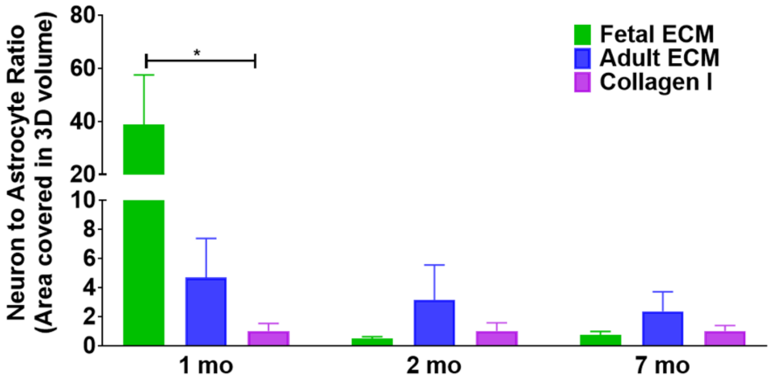

Supplementary Figure 1: Neuron to astrocyte ratio calculated by dividing the total volume in 3D confocal stacks covered by neurons versus astrocytes post basic image processing. One way ANOVA on log transformed data with Dunnett's posthoc at each time point, p= 0.0344, n=3-6.

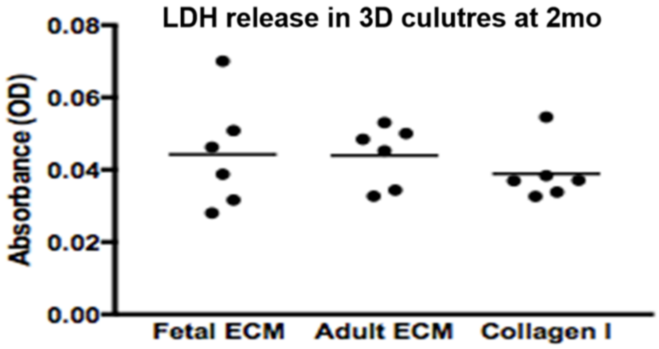

Supplementary Figure 2: Toxicity levels at 2 mo time point in hiNSC 3D cultures as measured by LDH release in media. No statistical difference (One-way ANOVA) in comparison to collagen I when the cells were grown in the presence of decellularized ECM.

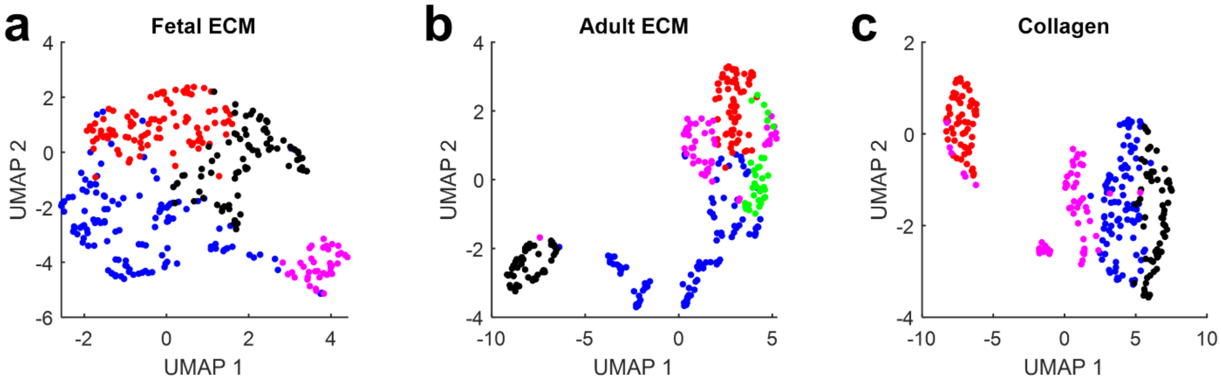

Supplementary Figure 3: Cluster Analysis at 3 months. UMAP plot of the ROIs from fetal ECM (a: *N*=341), adult ECM (b: *N*=294), and Collagen I (c: *N*=277) constructs at 3 months colored by five clusters. Clustering was conducted separately for each type of construct (*n*=3 cultures per type of construct). Cluster ID: 1 = blue dots; 2 = red dots; 3 = black dots; 4 = magenta dots; 5 = green dots

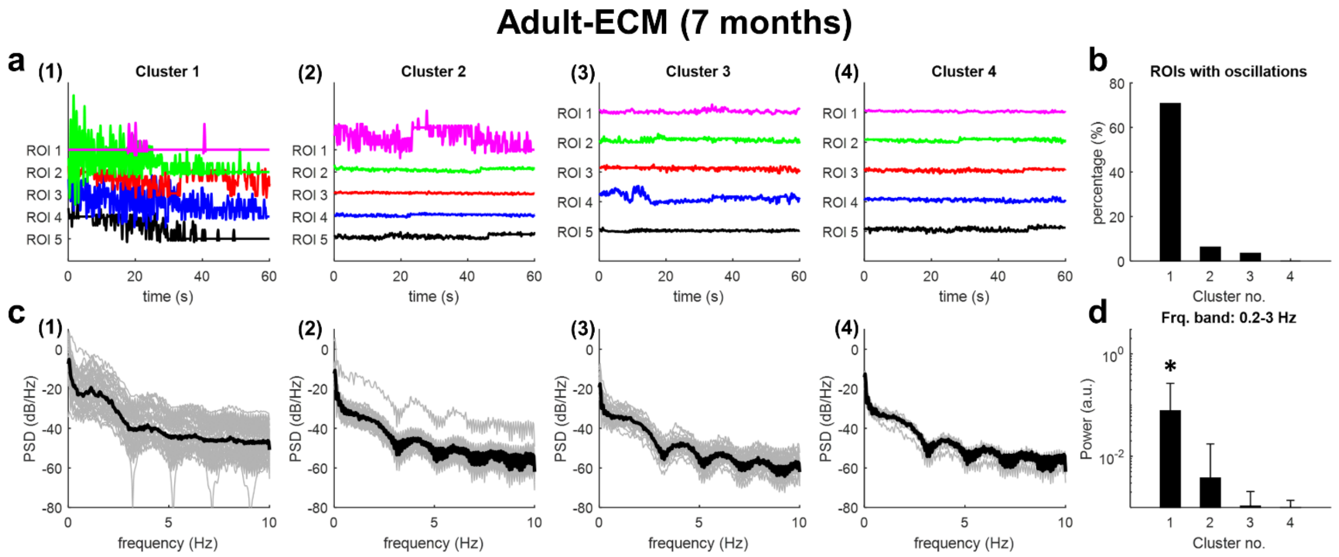

Supplementary Figure 4: Calcium activity from clusters in Adult-ECM constructs at 7 months. For cluster 1 through 4, panel a) reports fluorescence signals Δ*F*/*F* from five sample ROIs from the cluster, while panel c) reports the power spectrum density (PSD) of all ROIs in the cluster (gray lines, one line per ROI) and their median PSD (thick black line). Sub-panels (1), (2), (3), and (4) in a)-c) are for cluster 1, 2, 3, and 4, respectively. Number of ROIs per cluster are *M*=41 (cluster 1), 32 (cluster 2), 29 (cluster 3), and 10 (cluster 4), respectively. Panel b) reports the percentage of ROIs in each cluster whose Δ*F*/*F* time series exhibit significant oscillations. Panel d) reports the power in the frequency band [0.2, 3] Hz for Δ*F*/*F* signals in each cluster. Values are mean ± S.E.M. across *M* ROIs, with *M* as in a-c). Asterisks denote significant difference between clusters (one-way ANOVA with Tukey-Kramer *post hoc* test, *P*-value *P*<0.01).

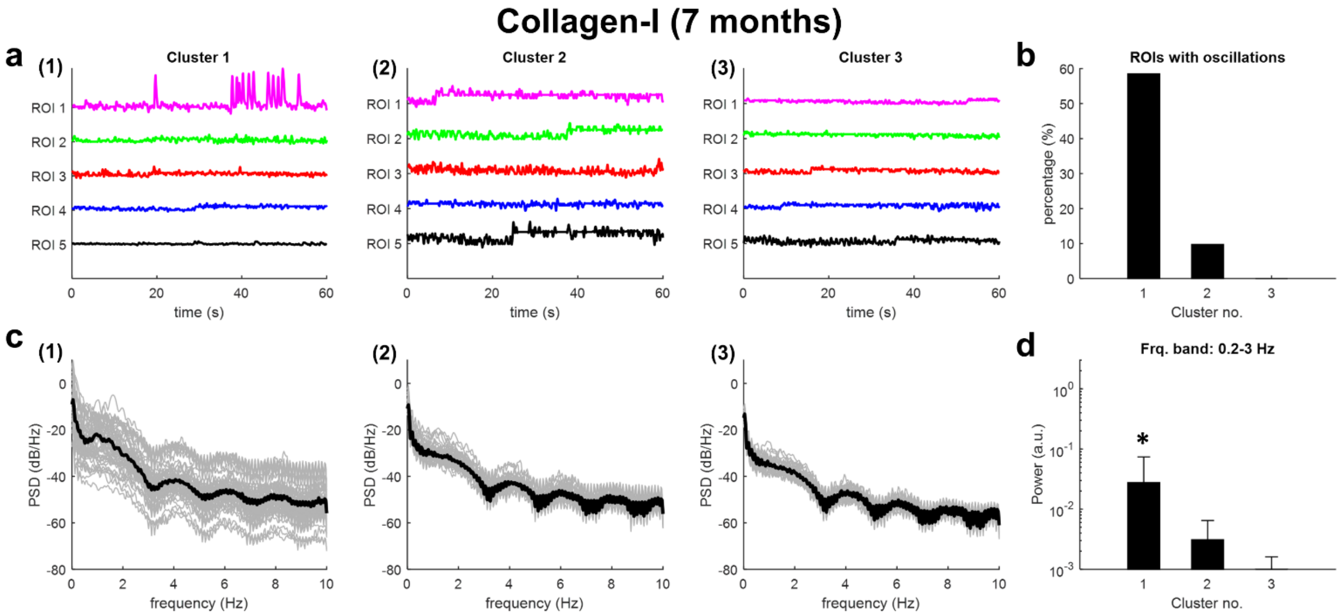

Supplementary Figure 5: Calcium activity from clusters in Collagen-I constructs at 7 months. For cluster 1 through 3, panel a) reports fluorescence signals Δ*F*/*F* from five sample ROIs from the cluster, while panel c) reports the power spectrum density (PSD) of all ROIs in the cluster (gray lines, one line per ROI) and their median PSD (thick black line). Sub-panels (1), (2), and (3) in a)-c) are for cluster 1, 2, and 3, respectively. Number of ROIs per cluster are *M*=53 (cluster 1), 41 (cluster 2), and 26 (cluster 3), respectively. Panel b) reports the percentage of ROIs in each cluster whose Δ*F*/*F* time series exhibit significant oscillations. Panel d) reports the power in the frequency band [0.2, 3] Hz for Δ*F*/*F* signals in each cluster. Values are mean ± S.E.M. across *M* ROIs, with *M* as in a-c). Asterisks denote significant difference between clusters (one-way ANOVA with Tukey-Kramer *post hoc* test, *P*-value *P*<0.001).

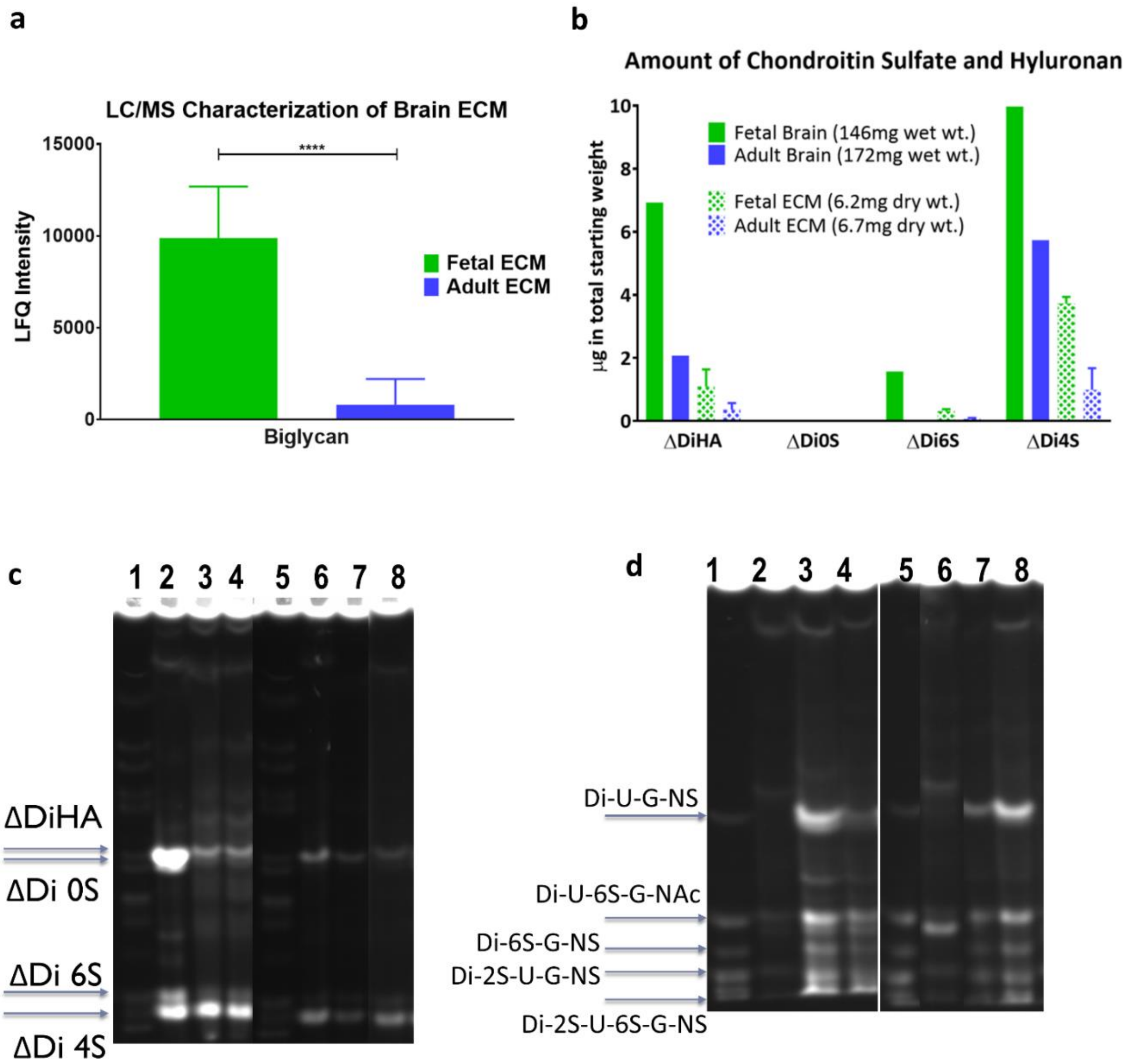

Supplementary Figure 6: **Characterization of decellularized porcine brain extracellular matrix**. (a) LFQ intensities corresponding to biglycan fragments observed in fetal versus adult porcine brain decellularized matrix, following LC/MS analysis. Unpaired two-tailed t-test, n=3, p=0.0073. (b) Overall higher quantity of GAGs present in fetal brain, with corresponding higher retention in the extracted fetal brain ECM based on FACE analysis. (c) Characterization of decellularized fetal & adult brain ECM in comparison to fetal and adult whole brains by FACE. Chondroitin sulfate (CS) and hyaluronan (HA) bands in the fetal & adult porcine brain ECM. Lanes 1/5,2,3/4,6,7/8 correspond to standard, fetal brain, fetal ECM, adult brain and adult ECM, respectively. (d) Heparin sulfate (HS) bands in the fetal & adult porcine brain ECM over multiple extractions. Abbreviations for HS disaccharides standards are as follows:

- delta DiHS - NS Heparan delta Di – NS:

2 - deoxy - 2 - sulfamino - 4 - O - (4 - deoxy - alpha - L - threo - hex - 4 -enopyranosyluronic acid) - D - glucose

- delta DiHS - 6S Heparan delta Di - 6S:

2 - acetamido - 2 - deoxy - 4 - O - (4 - deoxy - alpha - L - threo - hex - 4 -enopyranosyluronic acid) - 6 - O - sulfo - D - glucose

- delta DiHS - diS1 Heparan delta Di - di (6, N) S:

2 - deoxy - 2 - sulfamino - (4 - deoxy - alpha - L - threo - hex - 4 -enopyranosyluronic acid) - D - glucose

- delta DiHS - diS2 Heparan delta Di - di (U, N) S:

2 - deoxy - 2 - sulfamino - (4 - deoxy - 2 -O - sulfo - alpha - L - threo - hex - 4 -enopyrano - syluronic acid) - D - glucose

- delta DiHS - triS Heparan delta Di - tri (U, 6, N) S:

2 - deoxy - 2 - sulfamino - (4 - deoxy - 2 - O - sulfo - alpha - L - threo - hex - 4 -enopyranoslyuronic acid) - 6 - O - sulfo - D - glucose

Supplementary Videos

Videos 1-1: Spontaneous calcium activity in 3D cultures of differentiating human neural stem cells at 7 months; cells from a fetal ECM containing construct. Recorded at 20 frames per sec (fps), video shown at 50fps for 500 frames.

Videos 1-2: Spontaneous calcium activity in 3D cultures of differentiating human neural stem cells at 7 months; cells from an adult ECM containing construct. Recorded at 20 frames per sec (fps), video shown at 50fps for 500 frames.

Videos 1-3: Spontaneous calcium activity in 3D cultures of differentiating human neural stem cells at 7 months; cells from an unsupplemented construct. Recorded at 20 frames per sec (fps), video shown at 50fps for 500 frames.

Videos 1-4: Spontaneous calcium activity in 3D cultures of differentiating human neural stem cells at 3 months; cells from a fetal ECM containing construct. Recorded at 20 frames per sec (fps), video shown at 50fps for 500 frames.

Videos 1-5: Spontaneous calcium activity in 3D cultures of differentiating human neural stem cells at 3 months; cells from an adult ECM containing construct. Recorded at 20 frames per sec (fps), video shown at 50fps for 500 frames.

Videos 1-6: Spontaneous calcium activity in 3D cultures of differentiating human neural stem cells at 3 months; cells from an unsupplemented construct. Recorded at 20 frames per sec (fps), video shown at 50fps for 500 frames.

Supplementary Table 1: List of genes tested for release in 3D differentiating human neural stem cell cultures.

CACNA1D	Voltage-dependent L-type calcium channel subunit alpha-1D
CAMK2G	Calcium/calmodulin-dependent protein kinase type II subunit gamma
DLG4	Disks large homolog 4
EAAT1	Excitatory amino acid transporter 1
EAAT2	Excitatory amino acid transporter 2

ENO1	Enolase 1
FABP7	Fatty Acid Binding Protein 7
GABBR1	Gamma-aminobutyric acid type B receptor subunit 1
GAD1	Glutamate decarboxylase 1
GFAP	Glial fibrillary acidic protein
GPHN	Gephyrin
GRIA1	Glutamate receptor 1
GRIN1	Glutamate receptor ionotropic, NMDA 1
KCNB1	Potassium voltage-gated channel subfamily B member 1
KCND2	Potassium voltage-gated channel subfamily D member 2
MAP2	Microtubule associated protein 2
MEGF10	Multiple epidermal growth factor-like domains protein 10
NCS1	Neuronal calcium sensor 1
NES	Nestin
PAX6	Paired box protein Pax-6
SCN1A	Sodium channel protein type 1 subunit alpha
SCN3A	Sodium channel protein type 3 subunit alpha
SERPINA3	Peptidase inhibitor Serpina3n
SOX2	Transcription factor SOX-2
Stat3	Signal transducer and activator of transcription 3
SYN1	Synapsin 1
THBS1	Thrombospondin 1
THBS2	Thrombospondin 2
TNC	Tenascin
TUBB3	Beta-III tubulin

Supplementary Table 2: List of cytokines tested for release in 3D differentiating human neural stem cell cultures

ACRP30	Adiponectin
BDNF	Brain-derived neurotrophic factor
BMP2	Bone morphogenetic protein 2
b-NGF	beta- Nerve growth factor
bFGF	Basic fibroblast growth factor
C5a	Complement component 5a
CNTF	Ciliary neurotrophic factor
EGF	Epidermal growth factor
G-CSF	Granulocyte-colony stimulating factor
GDNF	Glial derived neurotrophic factor
GM-CSF	Granulocyte-macrophage colony-stimulating factor
GROa	Chemokine (C-X-C motif) ligand 1
ICAM-1	Intercellular Adhesion Molecule 1
IGF-1	Insulin growth factor-1
IL-10	Interleukin 10
IL-1a	Interleukin 1 alpha
IL-1b	Interleukin 1 beta
IL-1ra	Interleukin-1 receptor antagonist
IL-6	Interleukin 6
IL-8	Interleukin 8/Chemokine (C-X-C motif) ligand 8
LIF	Leukemia inhibitory factor
MCP-1	Chemokine ligand 2/CCL2
MIP-1 beta	Macrophage Inflammatory Protein-1
MIP-1a	Chemokine ligand 3/CCL3
MMP-2	Matrix metalloproteinase 2
MMP-9	Matrix metalloproteinase 9
NT-3	Neurotrophin-3

PDGF-AA	Platelet growth factor
RANTES	Chemokine ligand 5/CCL5
TGF beta 1	Transforming growth factor beta 1
TIMP-1	Tissue inhibitor of matrix metalloproteinase 1
TIMP-2	Tissue inhibitor of matrix metalloproteinase 2
TIMP-4	Tissue inhibitor of matrix metalloproteinase 4
TNFa	Tumor necrosis factor alpha
TSP-1	Thrombospondin 1
TSP-2	Thrombospondin 2
VEGF-A	Vascular endothelial growth factor A
